# Correction: Abdulrahim et al. Evaluation of Tissue Tropism and Horizontal Transmission of a Duck Enteritis Virus Vectored Vaccine in One-Day-Old Chicken. *Viruses* 2024, *16*, 1681

**DOI:** 10.3390/v16121931

**Published:** 2024-12-18

**Authors:** Yassin Abdulrahim, Yingying You, Linggou Wang, Zhixiang Bi, Lihua Xie, Saisai Chen, Benedikt B. Kaufer, Armando Mario Damiani, Kehe Huang, Jichun Wang

**Affiliations:** 1 College of Veterinary Medicine, Nanjing Agricultural University, Nanjing 210095, China; yassin1989@163.com (Y.A.); grace081606@163.com (L.W.); 2 Institute of Veterinary Immunology and Engineering, Jiangsu Academy of Agricultural Sciences, Nanjing 210014, China; hiyouyy@163.com (Y.Y.); zhixiangbi@163.com (Z.B.); lhxie999@163.com (L.X.); 13912965070@163.com (S.C.); 3 College of Veterinary Sciences, Nyala University, Nyala P.O. Box 155, South Darfur, Sudan; 4 College of Veterinary Medicine Shandong, Agricultural University, Taian 271018, China; 5 College of Animal Science and Technology, Guangxi University, Nanning 530004, China; 6 Institute of Virology, Freie Universität Berlin, Robert von Ostertag-Straße 7-13, 14163 Berlin, Germany; b.kaufer@fu-berlin.de; 7 Veterinary Centre for Resistance Research (TZR), Freie Universität Berlin, 14163 Berlin, Germany; 8 Laboratorio de Bioquímica e Inmunidad, Facultad de Ciencias Médicas, Universidad Nacional de Cuyo, Mendoza 5502, Argentina; 9 Instituto de Medicina y Biología Experimental de Cuyo, Consejo Nacional de Investigaciones Científicas y Técnicas (IMBECU-CONICET), Mendoza 5500, Argentina

## Addition of an Author

In the original publication [1], “Benedikt B. Kaufer” was not included as an author. The corrected Author Contributions statement appears here. 

Y.A., B.B.K. designed the experiment. Y.A. and Y.Y. performed the experiment, and Y.A., S.C. and L.W. collected the samples. Y.A., Y.Y. and L.W. processed all samples. Y.A. A.M.D. and B.B.K. analyzed the data, prepared the figures, and wrote the first draft of the manuscript. HVT-VP2-IBVD preparation was performed by L.X. The manuscript was critically reviewed by J.W., B.B.K., A.M.D. and K.H., supervision B.B.K. Z.B. completed HI and AGID tests. All authors have read and agreed to the published version of the manuscript.

## Additional Affiliations

Because of the additional author “Benedikt B. Kaufer”, 2 new affiliations 6 and 7 have been added. The updated affiliations should include “^6^ Institute of Virology, Freie Universität Berlin, Robert von Ostertag-Straße 7-13, 14163 Berlin, Germany; b.kaufer@fu-berlin.de; ^7^ Veterinary Centre for Resistance Research (TZR), Freie Universität Berlin, 14163 Berlin, Germany”. 

## Corrected Funding

Due to the same reasons, the funder “Jiangsu Academy of Agricultural Science”, “(JAAS2024-6)” to “Benedikt B. Kaufer” was not included. The corrected funding should read:

The support for the current work was provided by the Natural Science Foundation of Jiangsu Province (BK20131334) and Jiangsu Academy of Agricultural Science, (JAAS2024-6) for a study on the insertion sites of duck enteritis virus for foreign genes.

The authors state that the scientific conclusions are unaffected. This correction was approved by the Academic Editor. The original publication has also been updated.
